# Supplementary material for: The genome sequence of Glacies alpinata (Scopoli, 1763) (Lepidoptera: Geometridae)
Source: Wellcome Open Res. 2025 Jul 30;10:392. [Version 1] doi: 10.12688/wellcomeopenres.24664.1 (PMC12417988; doi:10.12688/wellcomeopenres.24664.1)
Supplement: Peer review history

# Reviewer report: Reviewer response for version 1

**DOI:** 10.21956/wellcomeopenres.27179.r128449

**Published:** 2025-09-08

**Authors:** Arun Arumugaperumal

**Affiliations:** 1 Department of Biotechnology, Rajalakshmi Engineering College, Thandalam ,Chennai, Tamil Nadu, India

The authors have presented the genome sequence of *Glacies alpinata*, a moth species. This is the first report of the high-quality genome sequence of the species. The assembly reported is of size 575.96 Mb. The genome sequence was distributed among 31 chromosomes. They have also assembled the mitogenome which is of size 17.2 kb. The links to the databases are working correctly. The authors have used high-end sequencing facility to sequence the genome which is of very high quality. The BUSCO completeness score of 98.6% is also an indicator of near-completeness of the genome assembly. The article may be indexed. However, the following typing errors need to be corrected.

 1. Change the typo in background section "in altitudes up to 3 000m"

 2. In methods section, under 'Nucleic acid extraction' correct the typo "and a yield of 1 311.30 ng"

Are sufficient details of methods and materials provided to allow replication by others?

Yes

Is the rationale for creating the dataset(s) clearly described?

Yes

Are the datasets clearly presented in a useable and accessible format?

Yes

Are the protocols appropriate and is the work technically sound?

Yes

Reviewer Expertise:

Bioinformatics; Genomics

I confirm that I have read this submission and believe that I have an appropriate level of expertise to confirm that it is of an acceptable scientific standard.

# Reviewer report: Reviewer response for version 1

**DOI:** 10.21956/wellcomeopenres.27179.r128450

**Published:** 2025-09-02

**Authors:** Annabel Whibley

**Affiliations:** 1 Grapevine Improvement, Bragato Research Institute, Lincoln, Lincoln, New Zealand

In this Data Note, Escuer and colleagues present a reference genome assembly of the Geometrid moth *Glacies alpinata.* Combining Revio HiFi data and Arima Hi-C, the obtained chromosomal reference haplotype assembly is of very high quality (6.C.Q66) with excellent completeness metrics. The templated report includes extensive QC including assembly statistics, a contact map, chromosome painting with Merian elements, k-mer profiling and contamination scanning with Blobtoolkit.  The report follows the standard template- methods are current and appropriate, the protocols and sample metadata are comprehensively documented in a well-structured report and public links to data accessions are functional.

 I will register here a query I expressed in an earlier review of a similar Data Note: the sample processing methods section contains hyperlinks to protocols which have been given DOIs. I assume that a new DOI would be minted when the protocol version is updated, but I wonder whether it would be helpful to also note the protocol versions in the text itself.

Are sufficient details of methods and materials provided to allow replication by others?

Yes

Is the rationale for creating the dataset(s) clearly described?

Yes

Are the datasets clearly presented in a useable and accessible format?

Yes

Are the protocols appropriate and is the work technically sound?

Yes

Reviewer Expertise:

Genomics, Bioinformatics

I confirm that I have read this submission and believe that I have an appropriate level of expertise to confirm that it is of an acceptable scientific standard.
